# Lithium prevents glucocorticoid‐induced osteonecrosis of the femoral head by regulating autophagy

**DOI:** 10.1111/jcmm.18385

**Published:** 2024-05-27

**Authors:** Qiuru Wang, Zhouyuan Yang, Qianhao Li, Wanli Zhang, Pengde Kang

**Affiliations:** ^1^ Department of Orthopedic Surgery, West China Hospital Sichuan University Chengdu China; ^2^ Public Laboratory Technology Center, West China Hospital Sichuan University Chengdu China

**Keywords:** autophagy, glucocorticoid, lithium, osteoblast, osteonecrosis of the femoral head

## Abstract

Autophagy may play an important role in the occurrence and development of glucocorticoid‐induced osteonecrosis of the femoral head (GC‐ONFH). Lithium is a classical autophagy regulator, and lithium can also activate osteogenic pathways, making it a highly promising therapeutic agent for GC‐ONFH. We aimed to evaluate the potential therapeutic effect of lithium on GC‐ONFH. For in vitro experiments, primary osteoblasts of rats were used for investigating the underlying mechanism of lithium's protective effect on GC‐induced autophagy levels and osteogenic activity dysfunction. For in vivo experiments, a rat model of GC‐ONFH was used for evaluating the therapeutic effect of oral lithium on GC‐ONFH and underlying mechanism. Findings demonstrated that GC over‐activated the autophagy of osteoblasts and reduced their osteogenic activity. Lithium reduced the over‐activated autophagy of GC‐treated osteoblasts through PI3K/AKT/mTOR signalling pathway and increased their osteogenic activity. Oral lithium reduced the osteonecrosis rates in a rat model of GC‐ONFH, and restrained the increased expression of autophagy related proteins in bone tissues through PI3K/AKT/mTOR signalling pathway. In conclusion, lithium can restrain over‐activated autophagy by activating PI3K/AKT/mTOR signalling pathway and up‐regulate the expression of genes for bone formation both in GC induced osteoblasts and in a rat model of GC‐ONFH. Lithium may be a promising therapeutic agent for GC‐ONFH. However, the role of autophagy in the pathogenesis of GC‐ONFH remains controversial. Studies are still needed to further explore the role of autophagy in the pathogenesis of GC‐ONFH, and the efficacy of lithium in the treatment of GC‐ONFH and its underlying mechanisms.

## 1 INTRODUCTION

Osteonecrosis of the femoral head (ONFH) is a common refractory orthopaedic disease, which tends to occur in young and middle‐aged people.^1^ In the United States, 30,000–60,000 new ONFH cases are diagnosed each year.^2^ At present, researchers generally believe that long duration or high doses of glucocorticoids (GC) is one of the main causes of ONFH.^3^ It is reported that about 9%–40% of people who receive high‐dose or long‐term GCs suffered from ONFH.^1^ ONFH can lead to the collapse of the femoral head, which is an irreversible process that affects the acetabulum. Therefore, the outcome of ONFH treatment can be improved by targeting the pre‐collapse stage.

Autophagy is a process in which eukaryotic lysosomes provide nutrients to cells by degrading damaged organelles, misfolded proteins, and intracellular pathogens.^4^ Autophagy may play an important role in the occurrence and development of glucocorticoid‐induced osteonecrosis of the femoral head (GC‐ONFH),^5^ but the exact mechanism remains controversial. Some studies have suggested that autophagy is a protective mechanism of the occurrence and development of GC‐ONFH.^6^, ^7^, ^8^, ^9^, ^10^ On the contrary, other studies have found that over‐activated autophagy can promote the occurrence and development of GC‐ONFH.^11^ PI3K/AKT/mTOR signalling pathway is one of the classic autophagy regulatory signalling pathways, and this pathway is currently the only known inhibitory pathway of autophagy.^12^ Activation of PI3K/AKT/mTOR signalling pathway can inhibit autophagy, while inhibition of this pathway induces autophagy.^12^

Lithium is a classical regulator of autophagy.^13^ Studies have reported that lithium can inhibit the apoptosis of bone marrow mesenchymal stem cells induced by serum deprivation or maintain the proliferation activity of liver cells by activating autophagy.^14^, ^15^ Lithium also exerts neuroprotective effects by regulating autophagy levels in mouse models of hypoxic–ischemic encephalopathy and Alzheimer's disease.^16^, ^17^ On the other hand, lithium can also activate osteogenic pathways. Studies have shown that lithium can promote osteoblast proliferation, differentiation and bone formation by activating the Wnt/β‐catenin signalling pathway.^18^, ^19^, ^20^ Therefore, lithium may be a highly promising therapeutic agent for GC‐ONFH. We assumed that lithium could promote healing of necrotic bone tissues in GC‐ONFH by regulating autophagy. We verified this hypothesis in vitro and in a rat model of GC‐ONFH.

## 2 MATERIALS AND METHODS

### 2.1 Experiments in vitro

The osteoblasts used in the present study were commercially available cells purchased from iCell Bioscience Inc. All osteoblasts used in the present study were primary osteoblasts obtained from rats (Art. no. RAT‐iCell‐s002; specifications: 5 × 10^5^), which were provided by iCell Bioscience Inc. The cells were cultured in specialized primary osteoblasts culture medium (Art. no. PriMed‐iCell‐019, iCell Bioscience Inc.).

#### 2.1.1 CYTO‐ID autophagy fluorescence staining

The CYTO‐ID Autophagy Detection Kit (Enzo Life Sciences, Inc.) detects autophagy vesicles using novel dyes that selectively label the accumulation of autophagy vesicles so that they exhibit bright fluorescence. Primary osteoblasts at logarithmic growth phase were prepared into single cell suspension. The cell density was adjusted to 1 × 10^5^ cells/ml and 1 mL of cell suspension was transferred into each well of 12‐well plate. Cells were treated with nothing (blank control) or dexamethasone (Beijing Solarbio Science & Technology Co., Ltd.) in different concentrations (125, 250 or 500 μM), with 4 replicates in each group. After the cells adhered to the walls, each well was added with the drugs corresponding to the aforementioned grouping, followed by incubation for 24 or 48 h at 37°C in an incubator with 5% CO_2_. Then, 300 μL of pre‐configured CYTO‐ID staining solution was added to each well, and stained at 37°C and 5% CO_2_ for 20 min. The cells were then observed and photographed under an inverted fluorescence microscope (Zeiss AG). Under 20x field of view, 5 fields of view were randomly selected, and the average fluorescence intensity was semi‐quantitatively calculated using Image J (version 1.8.0; US National Institutes of Health).

After screening suitable dexamethasone concentration (500 μM) and duration of action (48 h) (under these conditions, the autophagy level of osteoblasts changed most significantly), cells were treated with nothing, dexamethasone (500 μM) or dexamethasone (500 μM) combined with different concentrations of lithium chloride (MilliporeSigma) (0.01, 0.1, 1 or 10 mM). Then CYTO‐ID autophagy fluorescence staining was performed with the same methods.

#### 2.1.2 Transmission electron microscope (TEM)

Primary osteoblasts at logarithmic growth phase were prepared into single cell suspension. The cell density was adjusted to 1 × 10^5^ cells/ml and 2 mL of cell suspension was transferred into each well of three 6‐well plates. Cells were treated with nothing, dexamethasone (500 μM), or dexamethasone (500 μM) combined with lithium chloride (10 mM) for 48 h at 37°C in an incubator with 5% CO_2_, with 4 replicates in each group. The cells were washed with phosphate buffer saline (PBS) twice, digested and collected by trypsin, then centrifuged at 250 × ***g*** for 5 min at room temperature, the supernatants were discarded and 0.5% glutaraldehyde fixation solution was slowly added along the tube wall using a dropper. Following resuspension, the cell suspension was placed in a 4°C environment for 10 min for fixation, then the cell suspension was transferred to a 1.5 mL tip bottom EP tube and centrifuged at 12000 rpm for 10 min at 4°C. The supernatant was gently discarded, the precipitation was retained, and 3% glutaraldehyde fixed solution was slowly added with a dropper. The samples were then observed under a transmission electron microscope (JEM‐1400FLASH, JEOL, Ltd.). Five normal cells were randomly selected from each sample and autophagosomes in cytoplasm were counted. The average number of autophagosomes was compared between groups.

#### 2.1.3 Cell Counting Kit‐8 (CCK8) assay

To measure the cell viability, the cell density was adjusted to 3 × 10^4^ cells/ml and 100 μL of cell suspension was transferred into each well of a 96‐well plate. Cells were treated with nothing, dexamethasone (500 μM), or dexamethasone (500 μM) combined with lithium chloride (10 mM) for 48 h at 37°C in an incubator with 5% CO_2_, with 4 replicates in each group. The CCK‐8 reagent (Apexbio Technology LLC) was diluted 1:10 and 110 μL diluted CCK‐8 solution was added into each well. After 2 h, the optical density (OD) value of each well was examined at 450 nm according to the manufacturer's instructions. Cell viability was calculated as follows: Cell viability (%) = (OD value of observation group − OD value of zero adjustment group)/(OD value of control group − OD value of zero adjustment group) × 100%.

#### 2.1.4 Alkaline phosphatase (ALP) staining

The cell density was adjusted to 1 × 10^5^ cells/ml and 2 mL of cell suspension was transferred into each well of three 6‐well plates. Cells were treated with nothing, dexamethasone (500 μM), or dexamethasone (500 μM) combined with lithium chloride (10 mM) for 48 h at 37°C in an incubator with 5% CO_2_, with 4 replicates in each group. After fixing with 4% paraformaldehyde, the ALP dyeing solution (Shanghai Yisheng Biotechnology Co., Ltd.) configured according to the instructions was added. After incubation at room temperature for 5‐30 min away from light, the ALP staining solution was removed and washed with distilled water for one to two times to terminate the staining reaction. Then, the cells were observed and photographed under an inverted microscope (Leica). Under 20x field of view, 5 fields of view were randomly selected for each sample to calculate the average value. ImageJ (version 1.8.0; US National Institutes of Health) was used to calculate the percentage of ALP expression area (%).

#### 2.1.5 RNA isolation and reverse transcription‐quantitative PCR (RT‐qPCR)

The cell density was adjusted to 1 × 10^5^ cells/ml and 2 mL of cell suspension was transferred into each well of three 6‐well plates. Cells were treated with nothing, dexamethasone (500 μM), or dexamethasone (500 μM) combined with lithium chloride (10 mM) for 48 h at 37°C in an incubator with 5% CO_2_, with 4 replicates in each group to extract RNA. To evaluate the expression levels of osteogenic and autophagy related genes, total RNA was extracted from osteoblasts using TRIzol® reagent (Invitrogen; Thermo Fisher Scientific, Inc.) based on instructions provided by the manufacturer. Total RNA was then reverse‐transcribed using the PrimeScript™ RT reagent kit (Promega Corporation). Reactions were performed using a 20 μL final volume with 2 μL of cDNA and 10 μL of SYBR™‐Green PCR Master Mix (Promega Corporation). The nucleotide sequences of PCR primers are listed in Table 1.

**TABLE 1:** Nucleotide sequences of PCR primers.

Primer	Forward	Reverse
ACTB	5′‐CATCACTATCGGCAATGAGCGGTTCC‐3′	5′‐ACGCAGCTCAGTAACAGTCCGCCTA‐3′
AKT	5′‐AACGGCAGGAGGAGGAGACGATGGA‐3′	5′‐CTCGTTCATGGTCACACGGTGCTTGG‐3′
LC3B	5′‐CCGTCCTGGACAAGACCAAGTTCCT‐3′	5′‐ACACTCACCATGCTGTGCCCATTCA‐3′
mTOR	5′‐AGAGGACCAGCAGCACAAGCAGGAG‐3′	5′‐GCAGTGGTGGTGGCATTGGTGATGTT‐3′
RUNX2	5′‐GCTCCCCAACCGTTTTGAAT‐3′	5′‐CCAGATCACAACTGGGGAGT‐3′

Cycle threshold (Ct) values were obtained using Thermo Scientific PikoReal software (version 2.2; Thermo Fisher Scientific, Inc.). Relative expression was calculated using the 2^−△△CT^ method.

#### 2.1.6 Cell protein extraction and western blot analysis

The cell density was adjusted to 1 × 10^5^ cells/ml and 2 mL of cell suspension was transferred into each well of three 6‐well plates. Cells were treated with nothing, dexamethasone (500 μM), or dexamethasone (500 μM) combined with lithium chloride (10 mM) for 48 h at 37°C in an incubator with 5% CO_2_, with 4 replicates in each group to extract protein. RIPA buffer (Wuhan Servicebio Technology Co., Ltd.) was used to lyse osteoblasts on ice for 10 min. Then, the lysate mixture was then centrifuged at 12,000 rpm for 10 min at 4°C. The BCA Protein Assay kit (Beyotime Institute of Biotechnology) was used to measure the total protein concentration and 20 μg protein/lane was separated by SDS‐PAGE on a 10% or 12% gel. The separated proteins were subsequently transferred onto a PVDF membrane and blocked for 2 h at 25°C in a PBS solution containing 5% skim milk powder. Subsequently, the membranes were incubated overnight at 4°C with primary antibodies against LC3B (rabbit; cat. no. ab192890; Abcam), phosphorylated AKT (rabbit; cat. no. 310021; Chengdu Zen Bioscience Co., Ltd.), AKT (rabbit; cat. no. 382804; Chengdu Zen Bioscience Co., Ltd.), phosphorylated mTOR (mouse; cat. no. sc‐293,133; Santa Cruz Biotechnology, Inc.), mTOR (mouse; cat. no. sc‐517,464; Santa Cruz Biotechnology, Inc.), RUNX2 (rabbit; cat. no. GTX00792; GeneTex), and β‐tubulin (rabbit; cat. no. AF7011; Affinity Biosciences). After washing with PBST, the membranes were incubated with the secondary polyclonal goat anti‐rabbit/mouse HRP‐conjugated antibodies (cat. no. ZDR‐5306 and ZDR‐5307; OriGene Technologies, Inc.) at room temperature for 2 h. Finally, after washing the membranes with PBST, the protein bands were visualized using the ECL method (Torchlight Hypersensitive ECL Western HRP Substrate; cat. no. 17046; Chengdu Zen Bioscience Co., Ltd.) and the Quantity One software (version 4.6.6; Bio‐Rad Laboratories, Inc.) was used to analyse the results with β‐tubulin as the loading control.

### 2.2 Animals

The animal protocols in this study were approved by the Animal Care and Use Committee of West China Medical School, Sichuan University, and they complied with the “Guidelines on the humane use and care of laboratory animals” of the US National Institutes of Health. The experiments involved a total of 36 adult male Sprague–Dawley rats weighing 450–500 g (Chengdu Dashuo Biotechnology Co., Ltd.). Animals were housed for 6 weeks in the Animal Center of Sichuan University before experiments.

#### 2.2.1 Establishment of GC‐ONFH model and intervention

The 36 rats were randomly divided into three groups: control group, disease group, and lithium group. GC‐ONFH models were established in disease group and lithium group. The 24 rats were injected with lipopolysaccharide (10 μg/kg; MilliporeSigma) intraperitoneally twice, with 24 h between injections. At 24 h after the last injection, they were injected once a day intramuscularly with methylprednisolone (40 mg/kg; Pfizer Pharmaceuticals Ltd) for three consecutive days. Rats in the control group were injected with the same volumes of saline and otherwise treated in the same way as disease group and lithium group.

One tablet of lithium carbonate (0.25 g; Hunan Qianjin Xiangjiang Pharmaceutical Co., Ltd.) was thoroughly ground to uniform crushing, and 25 mL pure water was added to form a uniform suspension with a concentration of 10 mg/mL. 24 h after establishment of GC‐ONFH model, rats in the lithium group were given intragastric administration with lithium carbonate suspension (10 mg/kg) once a day, at an interval of 24 h for 8 weeks. Rats in the control group and disease group were given intragastric administration with 0.5 mL of normal saline every day.

#### 2.2.2 Sample collection

At 8 weeks after the last methylprednisolone injection, all animals were sacrificed, and the bilateral femoral heads were dissected. All the collected samples were washed twice with ice‐cold PBS, then one femoral head from each animal was randomly selected and fixed for 24 h in 10% formalin for further study such as micro‐computed tomography and histological study.

#### 2.2.3 Micro‐computed tomography

The femoral head was scanned using small‐animal micro‐computed tomography (PerkinElmer, Inc.) and 3D reconstruction was performed using the manufacturer's software. Scanning parameters were voltage, 90 kV; current, 88 A; resolution, 50 μm; and scanning time, 14 min. Trabecular bone parameters were assessed over the entire femoral head area. Bone volume/total volume (%) and trabecular number per mm were calculated automatically using the manufacturer's software.

#### 2.2.4 Haematoxylin–eosin staining

Femoral head tissue was sectioned and stained with haematoxylin–eosin (HE, Wuhan Servicebio Technology Co., Ltd.) in order to assess histomorphology, observe the phenomenon of empty osteocytes lacunae, and assess the extent of trabecular bone destruction as described.^21^, ^22^ Formalin‐fixed femoral heads were decalcified for 4 weeks in a 10% solution of ethylenediaminetetraacetic acid (EDTA), embedded in paraffin, cut into sections 3 μm thick, deparaffinized in xylene, and dehydrated in ethyl alcohol. Finally, the sections were stained with HE.

Animals were considered to have GC‐ONFH if their femoral head tissue showed at least one lesion with empty osteocyte lacunae or bone nucleus pyknosis, accompanied by necrosis of surrounding bone marrow cells.^23^ The incidence of GC‐ONFH were compared between groups.

#### 2.2.5 Goldner staining

Femoral head tissue was sectioned and stained with Goldner Staining Kit (MilliporeSigma) to detect the amount of osteoid (an organic matrix secreted by osteoblasts as part of new bone) in femoral head tissue according to the instructions provided by the manufacturer. After staining, mineralized bone appears green, osteoid appears orange or red, cartilage appears purple, and nucleus appears blue or grey. Under 20x field of view, 5 fields of view were randomly selected from the subchondral bone area for each sample to calculate the average value. Image J (version 1.8.0; US National Institutes of Health) was used to semi‐quantitatively analyse the proportion of osteoid area in the subchondral region of the femoral head.

#### 2.2.6 Immunohistochemistry

Femoral head tissue sections were subjected to immunohistochemistry using primary antibody against the proteins to examine the expression level of related proteins. Primary antibodies included LC3B (rabbit; cat. no. ab192890; Abcam), phosphorylated AKT (rabbit; cat. no. 310021; Chengdu Zen Bioscience Co., Ltd.), AKT (rabbit; cat. no. 382804; Chengdu Zen Bioscience Co., Ltd.), phosphorylated mTOR (mouse; cat. no. sc‐293133; Santa Cruz Biotechnology, Inc.), mTOR (mouse; cat. no. sc‐517464; Santa Cruz Biotechnology, Inc.), and RUNX2 (rabbit; cat. no. GTX00792; GeneTex). After incubation with primary antibody, sections were incubated with streptavidin‐biotin complex (Jackson ImmunoResearch). Primary antibody binding was detected using a DAB kit (Abcam), and sections were counterstained with haematoxylin. Under 20x field of view, 5 fields of view were randomly selected from the subchondral bone area for each sample to calculate the average value. Image J (version 1.8.0; US National Institutes of Health) was used to semi‐quantitatively analyse the proportion of positive expression area for these proteins in the subchondral region of the femoral head.

### 2.3 Statistical Analysis

Statistical analysis was performed using SPSS 26.0 (IBM, Chicago, IL, USA). For continuous data, we used one‐way analysis of variance (ANOVA) and performed post hoc testing using Tukey's test. For categorical data, we used Pearson's chi‐squared test or Fisher's exact probabilities test. Continuous data are presented as mean and standard deviation, unless otherwise indicated. Categorical data are presented as numbers or percentages. Significance was defined as *p* < 0.05.

## 3 RESULTS

### 3.1 Effects of GC and lithium on autophagy level of osteoblasts

**FIGURE 1:**
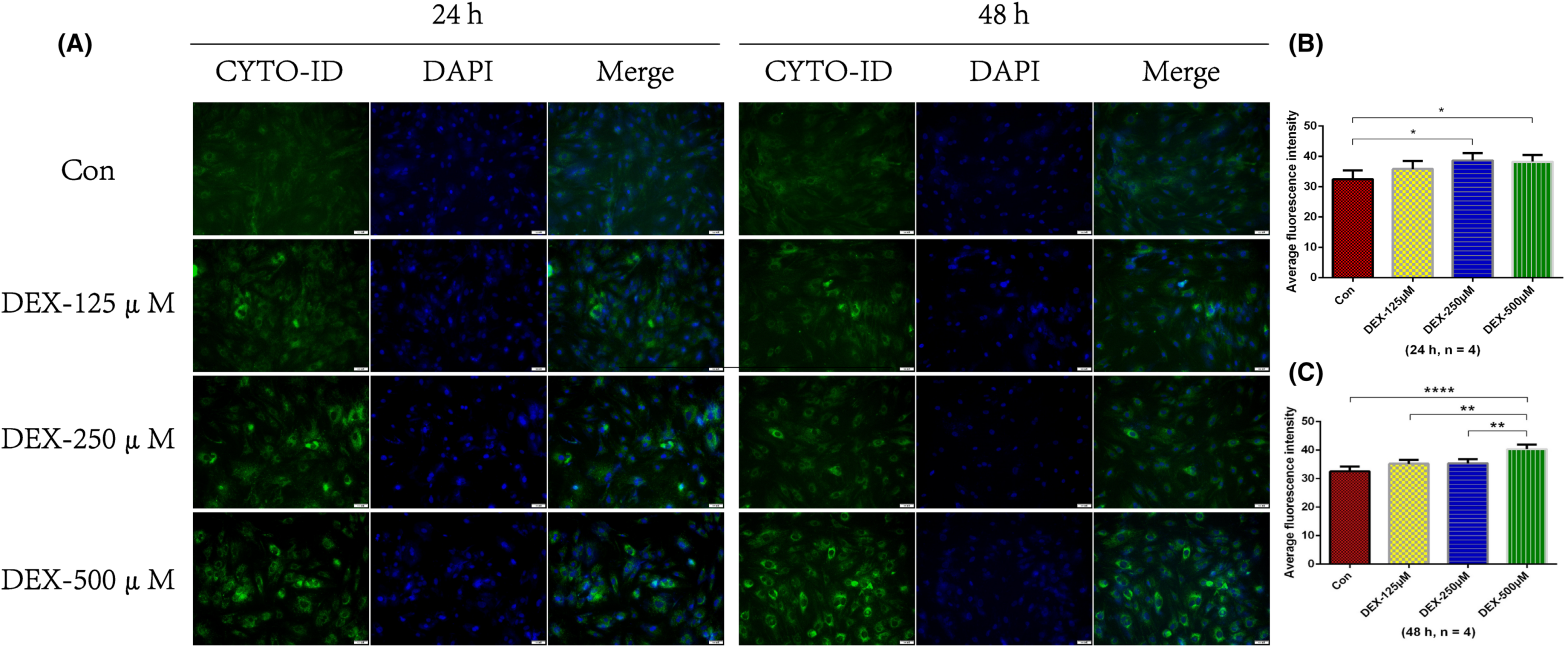
CYTO‐ID autophagy fluorescence staining of osteoblasts treated with nothing (control group) and dexamethasone in different concentrations (125 μM, 250 μM or 500 μM). (A) Typical images of CYTO‐ID autophagy fluorescence staining. Magnification, 200X. (B) The average fluorescence intensity of CYTO‐ID autophagy fluorescence staining at 24 h and (C) 48 h. *p < 0.05; **p < 0.01; ****p < 0.0001, compared with each other. The error bars indicate the standard deviation of the mean. Con: control group; DEX: dexamethasone group.

The results of CYTO‐ID autophagy fluorescence staining showed that the autophagy level of osteoblasts increased to varying degrees after treatment with dexamethasone of different concentrations (125, 250, or 500 μM) for 24 h or 48 h (Figure 1). However, after treated with 500 μM dexamethasone for 48 h, the autophagy level of osteoblasts increased most significantly.

**FIGURE 2:**
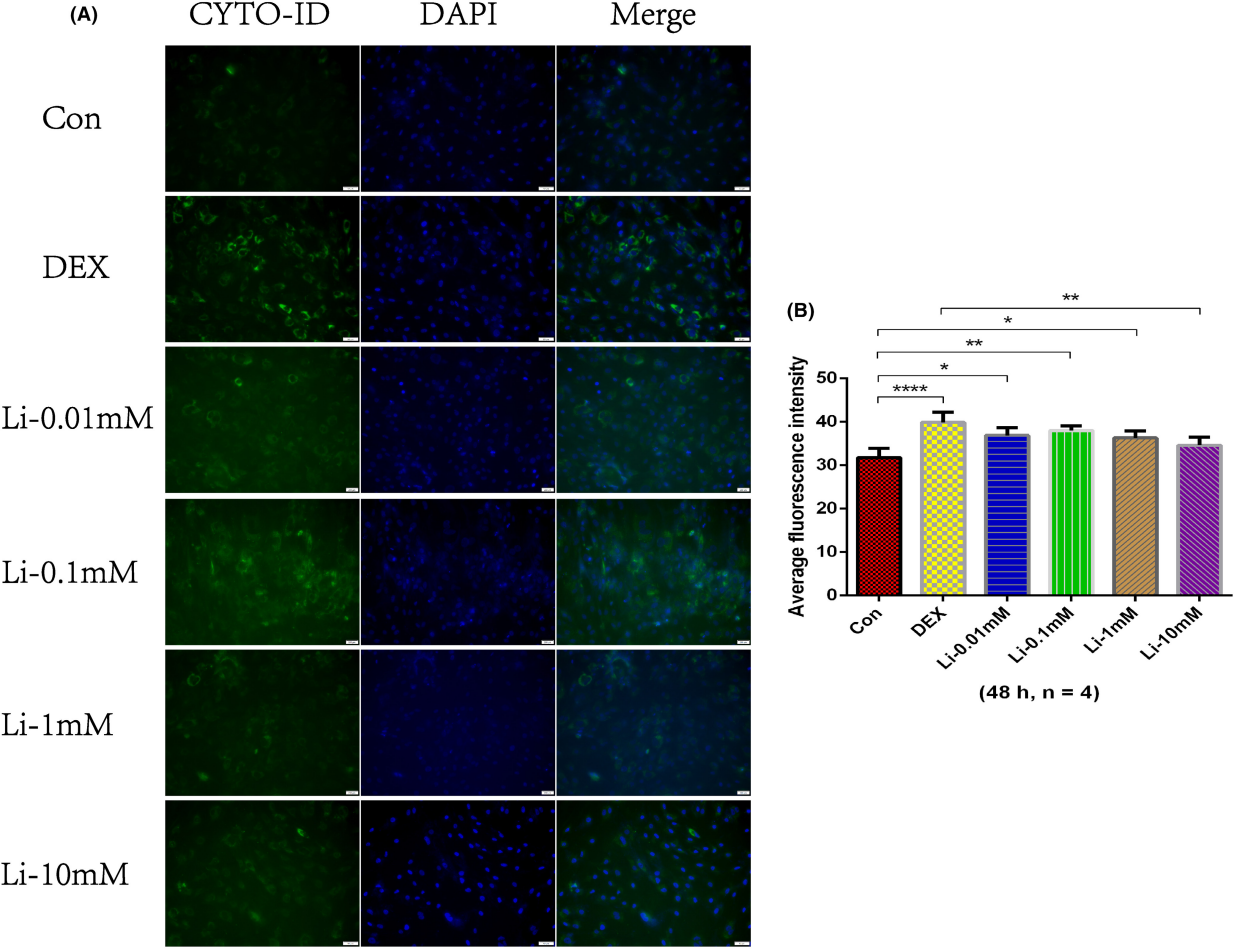
CYTO‐ID autophagy fluorescence staining of osteoblasts treated with nothing (control group), dexamethasone (500 μM) and dexamethasone (500 μM) combined with different concentrations of lithium chloride (0.01 mM, 0.1 mM, 1 mM or 10 mM). (A) Typical images of CYTO‐ID autophagy fluorescence staining. Magnification, 200X. (B) The average fluorescence intensity of CYTO‐ID autophagy fluorescence staining at 48 h. *p < 0.05; **p < 0.01; ****p < 0.0001, compared with each other. The error bars indicate the standard deviation of the mean. Con: control group; DEX: dexamethasone group; Li: dexamethasone combined with lithium chloride group.

After treatment with dexamethasone (500 μM) or dexamethasone (500 μM) combined with different concentrations of lithium chloride (0.01 mM, 0.1 mM, 1 mM or 10 mM) for 48 h, we found that 10 mM of lithium chloride could reduce the autophagy level overactivated by dexamethasone most significantly (Figure 2). Therefore, osteoblasts were divided into three groups in subsequent studies (control group: treated with nothing; dexamethasone group: treated with 500 μM of dexamethasone for 48 h; lithium group: treated with 500 μM of dexamethasone and 10 mM of lithium chloride for 48 h).

**FIGURE 3:**
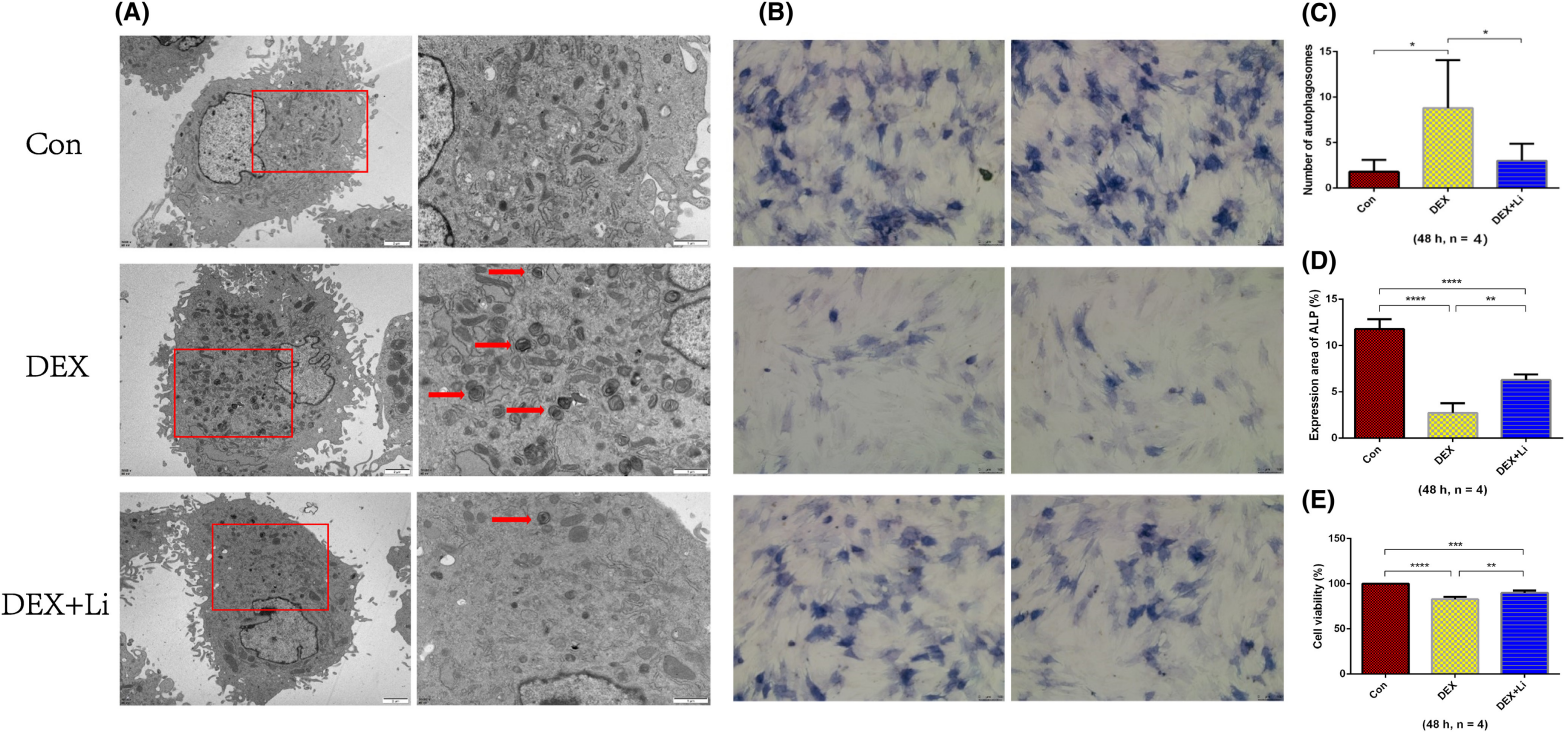
The results of transmission electron microscope (TEM), alkaline phosphatase (ALP) staining and cell counting kit‐8 (CCK8) assay. (A) Typical images of TEM. Boxes in the images at left (magnification, 6000X) show the areas enlarged on the right (magnification, 24,000X). Red arrows indicate autophagosomes. (B) Typical images of ALP staining. Magnification, 20X. (C) The average number of autophagosomes in each group. (D) The average percentage of ALP expression area in each group. (E) The average cell viability (CCK8 assay) in each group. *p < 0.05; **p < 0.01; ***p < 0.001; ****p < 0.0001, compared with each other. The error bars indicate the standard deviation of the mean. Con: control group; DEX: dexamethasone group (500 μM); DEX + Li: dexamethasone (500 μM) combined with lithium chloride (10 mM) group.

Compared with control group and lithium group, significantly more autophagosomes could be observed under TEM in the dexamethasone group (Figure 3).

### 3.2 Effects of GC and lithium on cell viability and ALP expression of osteoblasts

The results of CCK8 assay showed that the average cell viability of dexamethasone group was significantly lower than control group and lithium group (Figure 3). The average cell viability of lithium group was significantly lower than control group. The results of ALP staining showed that the average expressed percentage of ALP of dexamethasone group was significantly lower than control group and lithium group (Figure 3). The average expressed percentage of ALP of lithium group was significantly lower than control group.

### 3.3 Expression of genes related to osteogenesis and autophagy in osteoblasts

**FIGURE 4:**
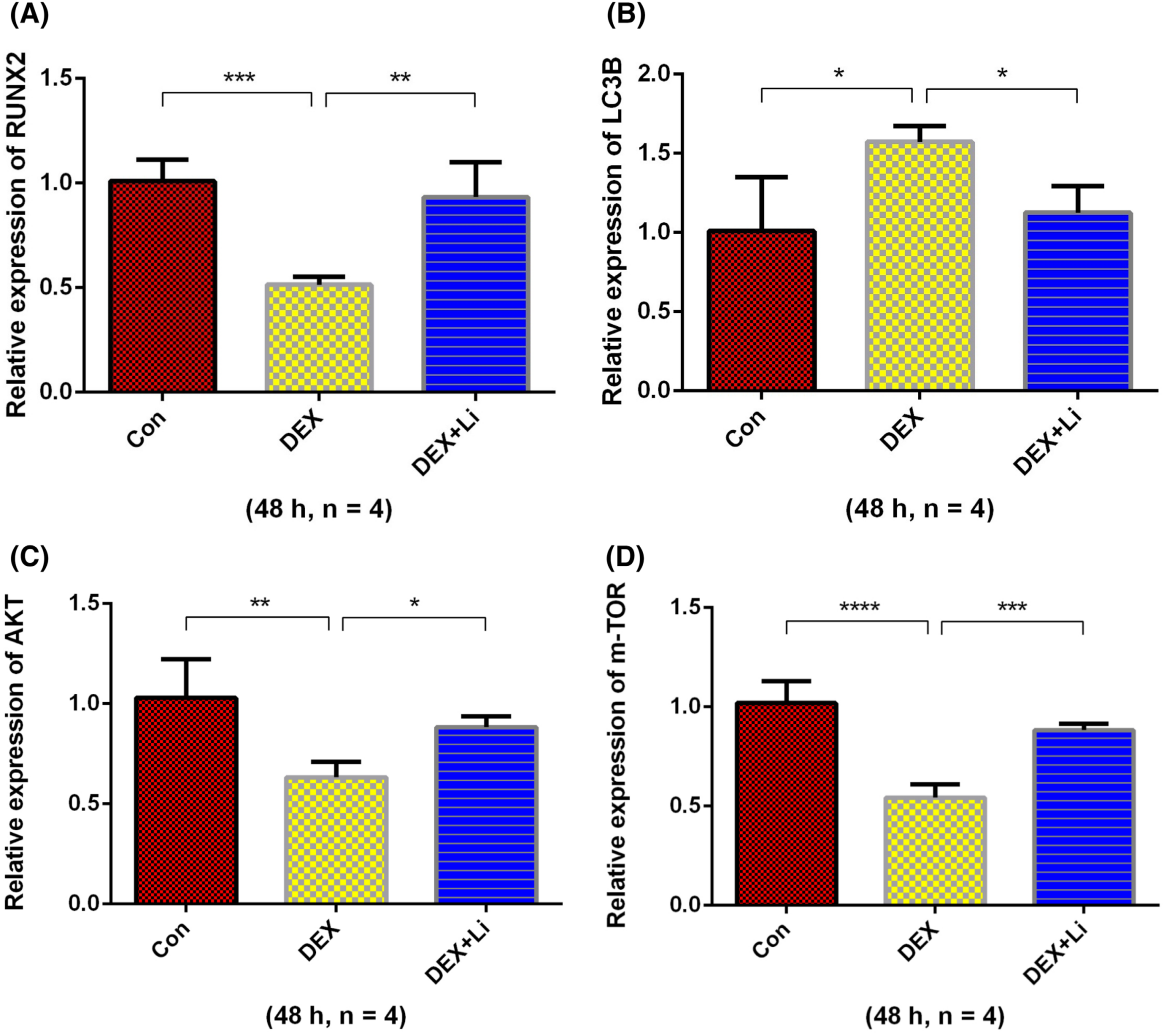
The results of RT‐qPCR from osteoblasts. The average relative expression of (A) RUNX2, (B) LC3B, (C) AKT, and (D) mTOR.

**FIGURE 5:**
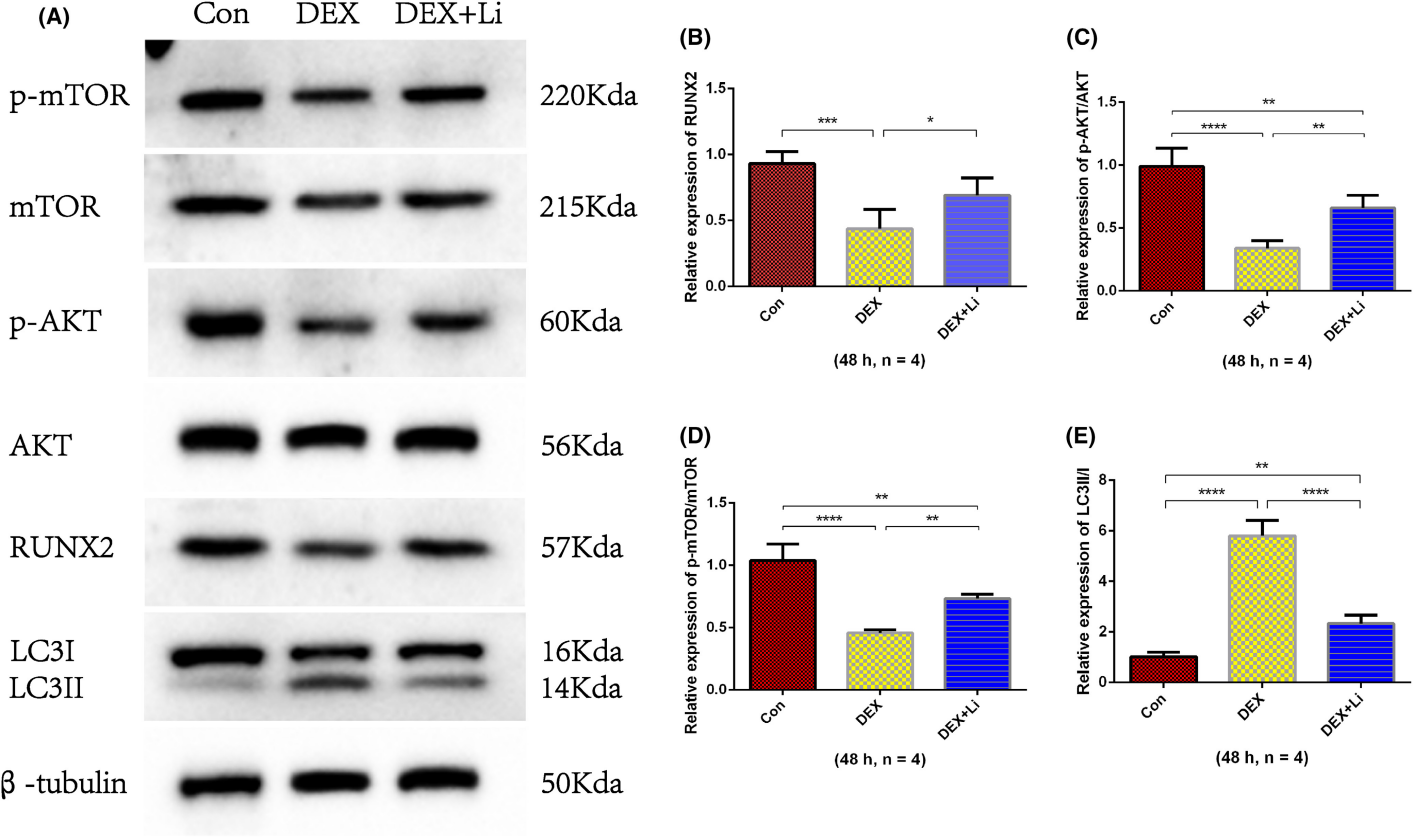
The results of western blot analysis from osteoblasts. (A) Typical images of western blot analysis from osteoblasts. The average relative expression of (B) RUNX2, (C) phosphorylated AKT/AKT, (D) phosphorylated mTOR/mTOR, and (E) LC3II/I. *p < 0.05; **p < 0.01; ***p < 0.001; ****p < 0.0001, compared with each other. The error bars indicate the standard deviation of the mean. Con: control group; DEX: dexamethasone group (500 μM); DEX + Li: dexamethasone (500 μM) combined with lithium chloride (10 mM) group. p‐AKT: phosphorylated AKT; p‐mTOR: phosphorylated mTOR.

The results of RT‐qPCR showed that the relative expression of RUNX2, AKT, and m‐TOR in dexamethasone group was significantly lower than control group and lithium group (Figure 4). The relative expression of LC3B in dexamethasone group was significantly higher than control group and lithium group. The results of western blot analysis of osteoblasts revealed that the relative expression of RUNX2, phosphorylated AKT/AKT, phosphorylated mTOR/mTOR in the dexamethasone group was significantly lower than that of the control and lithium groups, while the relative expression of LC3II/I in the dexamethasone group was significantly higher than that of the control and lithium groups (Figure 5).

### 3.4 Baseline data of animals

During the experiment, one rat in the control group died of unknown causes. The control group, disease group, and lithium group did not differ significantly from each other in body weight at baseline or at sacrifice (Table 2).

**TABLE 2:** Body weight of animals.

Time points	Control group (*n* = 11)	Disease group (*n* = 12)	Lithium group (*n* = 12)	*p* value
Weight before establishing model (g)	495.5 ± 38.4	504.1 ± 43.2	500.7 ± 23.0	0.847
Weight before sacrifice (g)	562.2 ± 33.7	555.6 ± 38.1	559.3 ± 32.5	0.902

### 3.5 Micro‐computed tomography of the femoral head

**FIGURE 6:**
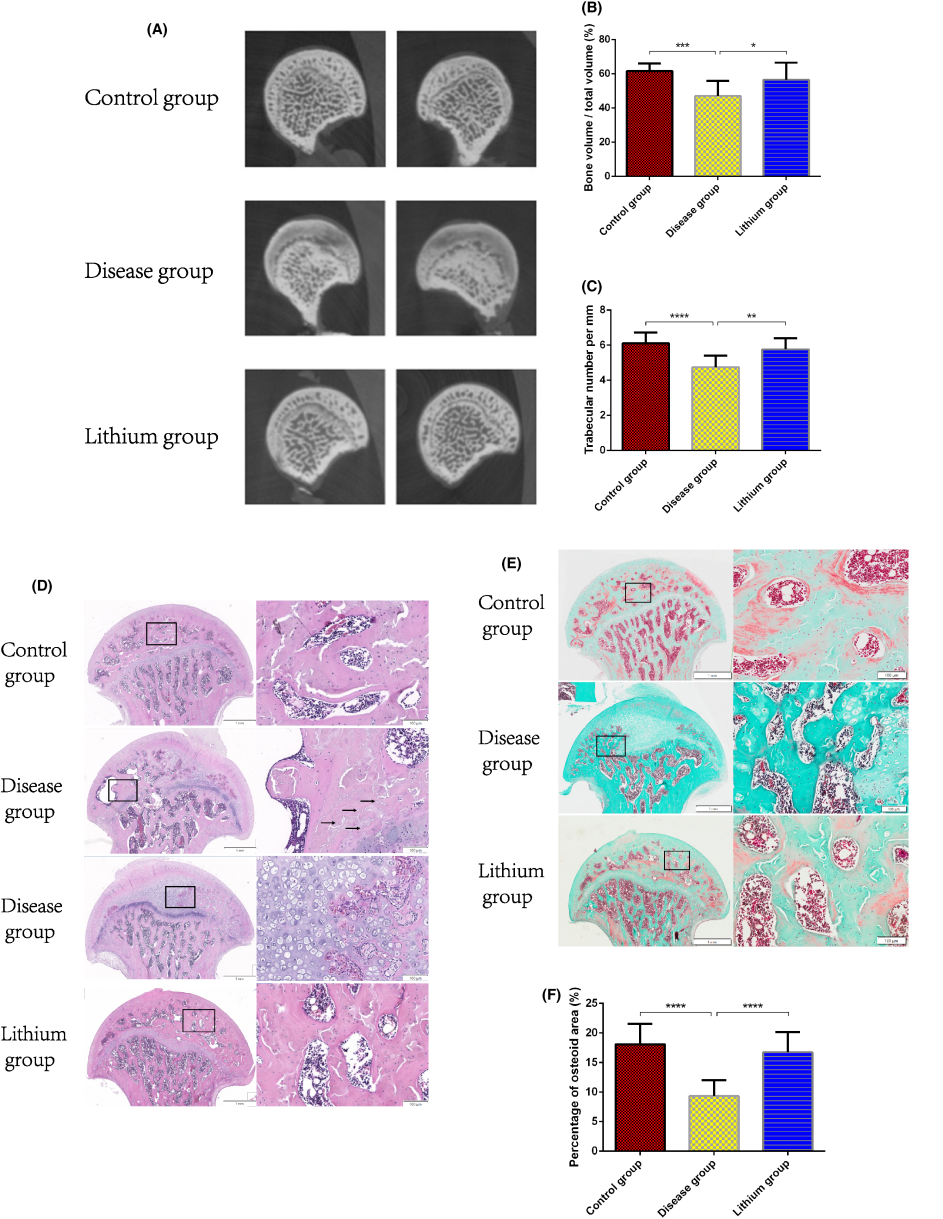
(A‐C) The results of micro‐computed tomography. (A) Typical images of micro‐computed tomography. (B) Bone volume as a percentage of total volume. (C) Trabecular number per mm. *p < 0.05; **p < 0.01; ***p < 0.001; ****p < 0.0001, compared with each other. The error bars indicate the standard deviation of the mean. (D‐F) The results of Haematoxylin–eosin staining and Goldner staining. The typical images of (D) Haematoxylin–eosin staining and (E) Goldner staining. Boxes in the images at left (magnification, 20X) show the areas enlarged on the right (magnification, 100X). Arrows in the second line of Haematoxylin–eosin staining indicate empty lacunae or pyknotic nucleus. The third line of Haematoxylin–eosin staining indicate abnormal hyperplasia of articular surface cartilage. (F) The average percentage of osteoid area of Goldner staining. ****p < 0.0001, compared with each other. The error bars indicate the standard deviation of the mean.

Compared with control group and lithium group, micro‐computed tomography showed significantly lower percentage of bone volume/total volume and significantly lower bone trabecular number in the disease group (Figure 6).

### 3.6 Histopathologic changes in the femoral head

HE staining of femoral head sections from the control group showed even staining, normal appearance of bone cells and their nuclei, regular bone trabecular arrangement with small spacing, and smooth articular surface cartilage (Figure 6). In contrast, tissue sections from the disease group showed typical signs of osteonecrosis, including many empty lacunae filling the bone trabeculae, disordered arrangement of bone trabeculae with widened spacing, some destroyed trabeculae, and necrosis of bone marrow cells. In addition, the articular surface cartilage showed abnormal hyperplasia that had replaced the subchondral trabecular area. Nuclei in areas of abnormal hyperplasia appeared pyknotic and denaturated. The histopathologic changes of femoral head in lithium group were similar to those in control group.

The results of Goldner staining showed that the area fraction of osteoid in the subchondral region of the femoral head was significantly lower in the disease group than in the control group and lithium group (Figure 6).

### 3.7 The necrosis rates of femoral head tissue

The necrosis rates were determined by histopathologic staining. The necrosis rates were 0/11 (0%) in the control group, 10/12 (83.3%) in the disease group, and 4/12 (33.3%) in the lithium group. Compared with disease group, the necrosis rates were significantly lower in the control group (*p* < 0.001) and lithium group (*p* = 0.013).

### 3.8 Expression of proteins related to osteogenesis and autophagy in the femoral head

**FIGURE 7:**
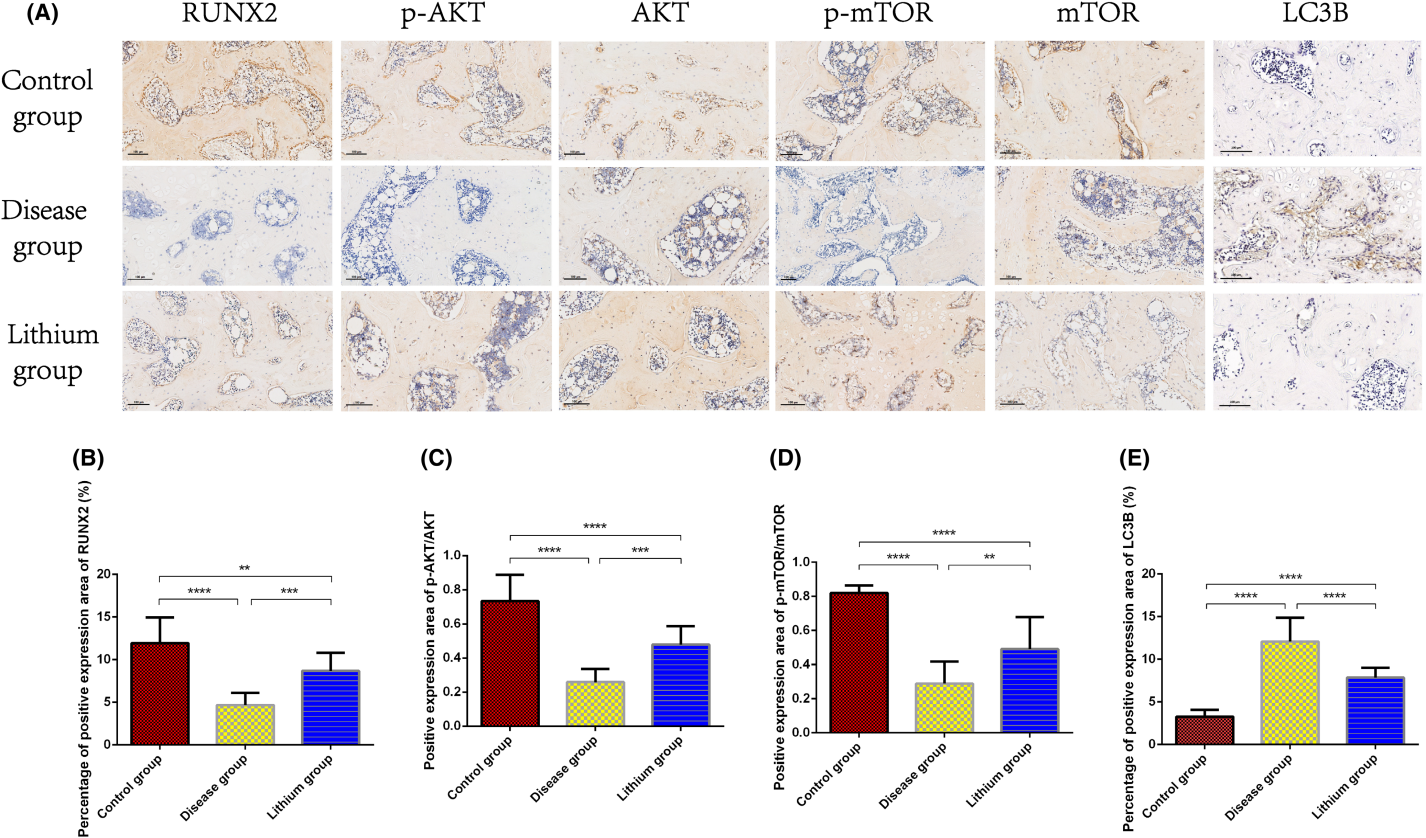
The results of immunohistochemistry. (A) Typical images of immunohistochemistry. Magnification, 200X. The average relative expression of (B) RUNX2, (C) phosphorylated AKT/AKT, (D) phosphorylated mTOR/mTOR, and (E) LC3B. **p < 0.01; ***p < 0.001; ****p < 0.0001, compared with each other. The error bars indicate the standard deviation of the mean. p‐AKT: phosphorylated AKT; p‐mTOR: phosphorylated mTOR.

The results of immunohistochemistry showed that the expression level of proteins in femoral head including RUNX2, phosphorylated AKT/AKT, phosphorylated mTOR/mTOR in disease group was significantly lower than control group and lithium group (Figure 7). The expression level of these proteins was significantly lower in lithium group compared with control group. The expression level of LC3B in disease group was significantly higher than control group and lithium group. The expression level of LC3B was significantly higher in lithium group compared with control group.

## 4 DISCUSSION

The most important finding of the present study was that after treated with dexamethasone 500 μM for 48 h, the autophagy level of osteoblasts was increased while the cell viability and osteogenic activity were decreased. Simultaneous intervention with 10 mM lithium chloride can reverse this phenomenon. In the rat GC‐ONFH model, the expression of autophagy markers in femoral head tissue was also increased. After 8 weeks of oral administration of lithium (10 mg/kg), the expression level of autophagy markers in femoral head tissue decreased, and the symptoms of femoral head necrosis were alleviated. In addition, we also found that lithium inhibits GC‐induced over‐activated autophagy by activating the AKT/m‐TOR signal pathway.

At present, researchers have proposed a variety of theories on the pathophysiological mechanism of GC‐ONFH, including lipid metabolism disorder theory, osteocyte apoptosis theory, osteoporosis theory, intraosseous hypertension theory, coagulation mechanism change theory and so on.^24^, ^25^, ^26^, ^27^ Although these theories are based on some basic experiments and clinical studies, they cannot completely explain the whole pathophysiological process of GC‐ONFH. Recent studies suggest that autophagy also plays an important role in GC‐ONFH.^5^, ^6^, ^7^, ^8^, ^9^, ^10^, ^11^

In this study, GC can increase LC3B expression in osteoblasts and femoral head tissues. LC3B is a marker protein of autophagosome, and its increased expression can be used to indicate the activation of autophagy.^28^, ^29^ Therefore, we speculate that over‐activated autophagy may be the inducement of ONFH induced by GC. Some previous researches also support our hypothesis. First, researchers found increased expression of autophagy marker proteins in femoral head tissue samples from patients diagnosed with GC‐ONFH.^11^, ^30^ Next, the researchers conducted cell experiments for further verification. Li et al reported that the treatment of mouse osteoblasts with dexamethasone decreased cell viability, and reduced the ALP and bone morphogenetic protein‐2 expression in osteoblasts in vitro. By contrast, autophagy inhibitor (3‐MA) treatment attenuated the cell injury induced by dexamethasone.^11^ In addition, Zhao et al reported that the autophagy flux in the rabbit osteoblasts increased and the ciliary length decreased in a time‐dependent manner after methylprednisolone treatment, so they speculated methylprednisolone inhibited primary cilia formation and promoted autophagy, which could be the pathological basis of GC‐ONFH.^31^ PENG et al.'s study also showed that the level of autophagy marker proteins was also significantly increased during the process of rat osteoblasts apoptosis induced by dexamethasone, while autophagy inhibitor 3‐MA attenuated dexamethasone‐mediated apoptosis.^32^

Both cell and animal experiments in this study demonstrated that lithium, as an autophagy regulator and osteogenic activator, can inhibit the excessive autophagy of osteoblasts induced by GC, while retaining the osteogenic activity of osteoblasts, and achieve the purpose of treating GC‐ONFH in rats. The recommended dose of lithium carbonate for clinical treatment is 20‐25 mg/kg/day. As the therapeutic dose of lithium is close to the toxic dose,^33^, ^34^ the dose used in this study is 10 mg/kg/day. Our results suggest that lithium may inhibit autophagy overactivated by GC by activating the PI3K/AKT/mTOR signalling pathway. PI3K/AKT/mTOR is an important signalling pathway that regulates autophagy, and is currently the only known inhibitory pathway that regulates autophagy. Autophagy can be inhibited after activation of this pathway.^12^ Known as protein kinase B, AKT becomes active when phosphorylated and then phosphorylates the mTOR protein. mTOR is an important substrate of AKT that senses and responds to various signals in eukaryotes to regulate biological processes such as cell growth and survival.^35^, ^36^ Studies have shown that mTOR is the main regulator of autophagy, and activation of mTOR (phosphorylation) can inhibit autophagy; conversely, inhibition of mTOR can induce autophagy.^37^

In addition to regulating autophagy, previous studies have demonstrated that lithium can promote osteoblast differentiation and inhibit lipid differentiation of bone marrow mesenchymal stem cells (BMSCs),^38^, ^39^ or improve osteoblast activity of osteoblast^40^ by activating the Wnt/β‐catenin signalling pathway. Researchers have also found that various biomaterials doped with lithium can also promote bone repair.^18^, ^19^, ^20^, ^41^, ^42^, ^43^ Lithium may be a promising drug for the prevention and treatment of GC‐ONFH due to its ability of both autophagy regulation and osteogenesis. According to our results, lithium can reverse or prevent osteonecrosis in GC‐ONFH model of rats. In the present study, lithium was administered just from 24 h after establishment of GC‐ONFH model for 8 weeks. Therefore, in clinical practice, starting lithium therapy immediately after receiving a high‐dose glucocorticoids intervention may be the prefer chance to reverse or prevent osteonecrosis. However, the optimal chance to reverse or prevent osteonecrosis with lithium is still unclear, and further studies are needed to explore this issue. Overall, the present study provided a preliminary theoretical basis for the prevention and treatment of GC‐ONFH with lithium.

This study creatively tried to prevent and treat GC‐ONFH in rats by regulating autophagy with lithium. However, autophagy is a complex physiological process. Currently, the role of autophagy in the pathogenesis of GC‐ONFH remains controversial. First, this study and some previously mentioned studies suggest that over‐activated autophagy may be the inducement of GC‐ONFH.^11^, ^30^, ^31^, ^32^ In contrast, some studies suggest that autophagy is a GC‐induced self‐protection mechanism in cells. Studies have shown that GC‐ONFH may be related to osteoblast apoptosis, and increasing the autophagy level of osteoblasts can prevent GC‐induced osteoblast apoptosis.^6^, ^7^, ^8^, ^9^, ^44^ In addition, pravastatin has been found to enhance autophagy of endothelial progenitor cells, thereby reducing the risk of GC‐ONFH.^45^ Therefore, much more work is needed in the future to further clarify the role of autophagy in the pathogenesis of GC‐ONFH.

This study only explored the osteoblasts in vitro. However, autophagy is a process that occurs in various eukaryotic cells. In addition to osteoblasts, GC also affect autophagy levels of other cells in the femoral head tissue, including BMSCs,^46^ osteocytes,^10^, ^47^, ^48^ osteoclasts,^49^ and chondrocytes.^50^ Moreover, the change of autophagy level induced by GC was related to the dose and duration of action. One previous study found that with the increase of dexamethasone dose, the autophagy level decreased after reaching the peak, and caused a decrease in the viability and number of osteocytes.^10^ In the early stage, cells rely on autophagy to adapt to a certain metabolic pressure, and in the presence of higher or sustained metabolic pressure, the production of excess autophagosomes induces the decline of cell vitality and functional changes. Under GC induction, the autophagy levels of various cells including BMSCs, osteoblasts, osteocytes, osteoclasts, and chondrocytes in bone tissue will change, and the changes in autophagy levels depend on the dosage and intervention duration of GC. Therefore, it is hard to present when and how autophagy happened of GC‐ONFH in vivo. In this study, we only observed the increased expression of autophagy related proteins in the femoral head tissue of GC‐ONFH rats. Other studies have also obtained similar results and found that the severity of GC‐ONFH was positively correlated with the level of autophagy protein expression.^6^, ^51^ However, in the process of GC‐ONFH, exactly which cells undergo changes in autophagy levels and when is still unclear. Studies are needed to further explore when and how autophagy happened in vivo during the occurrence and development of GC‐ONFH.

In our vitro experiments, lithium chloride could reverse GC‐induced autophagy directly on osteoblasts. However, lithium chloride may not only affect autophagy levels in osteoblasts in vivo. Although no studies have directly explored the effect of lithium on autophagy levels in other cells in GC‐ONFH, one study has reported that lithium chloride can regulate autophagy level of BMSCs in osteoporotic model of rats.^52^ Therefore, the change in autophagy levels of osteoblasts may be the adjoint result of lithium on rats. Lithium may indirectly regulate autophagy levels in osteoblasts by regulating the function of other cells in vivo. Furthermore, lithium may also promote healing of necrotic bone tissues in rats through other mechanisms and the changes in autophagy levels in bone tissue may be just the adjoint result of lithium on rats. Future studies are still needed to explore the efficacy of lithium in the treatment of GC‐ONFH and its underlying mechanisms.

In addition, the results of this study are based on rat cells and model, GC‐ONFH model of rats could not totally represent the disease situation of human beings. Although lithium treatment could significantly reduce the necrosis rates of GC‐ONFH model in rats, further studies are needed to confirm whether the results of this study can be applied to human beings.

In conclusion, lithium can restrain over‐activated autophagy by activating PI3K/AKT/mTOR signalling pathway and up‐regulate the expression of genes for bone formation both in osteoblasts and in a rat model of GC‐ONFH. Lithium may be a promising therapeutic agent for GC‐ONFH. However, the role of autophagy in the pathogenesis of GC‐ONFH remains controversial. Studies are still needed to further explore the role of autophagy in the pathogenesis of GC‐ONFH, and the efficacy of lithium in the treatment of GC‐ONFH and its underlying mechanisms.

## AUTHOR CONTRIBUTIONS

**Qiuru Wang:** Data curation (equal); investigation (equal); methodology (equal); writing – original draft (equal). **Zhouyuan Yang:** Conceptualization (equal); investigation (equal); methodology (equal); writing – original draft (equal). **Qianhao Li:** Data curation (equal); investigation (equal); writing – original draft (equal). **Wanli Zhang:** Investigation (equal); software (equal); writing – original draft (equal). **Pengde Kang:** Conceptualization (equal); methodology (equal); writing – review and editing (equal).

## CONFLICT OF INTEREST STATEMENT

The authors declare no conflict of interest.

## Data Availability

The datasets generated for this study are available on request from the corresponding author.
